# Low-Cost Robotic Guide Based on a Motor Imagery Brain–Computer Interface for Arm Assisted Rehabilitation

**DOI:** 10.3390/ijerph17030699

**Published:** 2020-01-21

**Authors:** Eduardo Quiles, Ferran Suay, Gemma Candela, Nayibe Chio, Manuel Jiménez, Leandro Álvarez-Kurogi

**Affiliations:** 1 Instituto de Automática e Informática Industrial, Universitat Politècnica de València, 46022 València, Spain; nachch@posgrado.upv.es; 2 Departament de Psicobiologia, Facultat de Psicologia, Universitat de València, 46010 València, Spain; ferran.suay@uv.es (F.S.); gemma.candela@uv.es (G.C.); 3 Facultad de Ingeniería, Ingeniería Mecatrónica, Universidad Autónoma de Bucaramanga, Bucaramanga 680003, Colombia; 4 Facultad de Educación, Universidad Internacional de la Rioja, 26006 Logroño, Spain; manuel.jimenez@unir.net (M.J.); leandro.alvarez@unir.net (L.Á.-K.)

**Keywords:** robotic rehabilitation, robot-assisted therapy, brain computer interfaces in neurorehabilitation, EEG sensors

## Abstract

Motor imagery has been suggested as an efficient alternative to improve the rehabilitation process of affected limbs. In this study, a low-cost robotic guide is implemented so that linear position can be controlled via the user’s motor imagination of movement intention. The patient can use this device to move the arm attached to the guide according to their own intentions. The first objective of this study was to check the feasibility and safety of the designed robotic guide controlled via a motor imagery (MI)-based brain–computer interface (MI-BCI) in healthy individuals, with the ultimate aim to apply it to rehabilitation patients. The second objective was to determine which are the most convenient MI strategies to control the different assisted rehabilitation arm movements. The results of this study show a better performance when the BCI task is controlled with an action–action MI strategy versus an action–relaxation one. No statistically significant difference was found between the two action–action MI strategies.

## 1. Introduction

Motor deficiencies are a great handicap for many disabled people. Such disabilities are caused by various medical problems (stroke, trauma, neurodegenerative diseases, etc.) and affect millions of people worldwide. After suffering a motor disability, the rehabilitation of motor function becomes a necessity. Rehabilitation of hands and arms is fundamental to improve the independence of affected patients when performing everyday tasks [1,2]. Many of the rehabilitation strategies applied, such as active motor training (AMT) [3], force the use of paralyzed limbs. AMT can produce an improvement in motor performance and a cortical reorganization. However, AMT usefulness is limited since it depends on a patient’s residual motor activity. This excludes many patients from the potential benefits of AMT.

Motor imagery (MI) may be an alternative rehabilitation strategy. MI can rely on the imagination of body movements and does not depend on parasitic or residual motor abilities [4,5,6]. Various studies point to the positive effect that motor imagination training can produce on motor recovery [7,8].

Brain–computer interfaces (BCIs) are communication/control systems that can be employed to transform the user’s intention into different actions by means of brain activity [9,10,11,12]. BCIs include sensors that record brain activity and software that processes this information in order to interact with the environment by means of actuators. Patients with limited communication and movement capabilities can benefit from this technology, which includes communication protocols such as spellers [13], control of robot arms [14] and neuro-prosthesis [15], control of motorized wheelchairs [16], and home automation systems [17]. Although BCI technology has attracted extensive research interest for several decades, it has not yet become a common technology in medical protocols [18]. Barriers that must be removed before BCI technology is ready for commercial purposes include individual customization of BCI applications (i.e., the need for individual and recurrent calibration, standardization of protocols and procedures, as well as patient convenience and comfort in using BCI devices such as electroencephalogram (EEG) electrodes and caps) [19]. Some research is currently intended to solve problems that prevent the generalization of medical and recreational BCI use [11,19,20]. The success of BCI technology will depend on improving its reliability and accuracy, i.e., to increase the number of times in which the system actually performs the intended action.

BCI technology can also be used to assist in rehabilitation, since BCIs aim to translate the patient’s MI into external commands to control a helping device. The thought or realization of a motor action is generated in the sensory–motor areas of the cerebral cortex, which causes a variation in the EEG signal [21]. Specifically, imagining motor actions usually modifies the amplitude of the mu/beta rhythms in the sensory–motor cortex [22]. These variations in the spectral content of EEG signals can be used to control a BCI system [23,24].

Non-invasive EEG-based BCI systems have been used to control robotic systems that can improve the daily life of affected individuals, as well as for the rehabilitation and recovery of motor skills [25]. A quadriplegic individual who only had some left bicep mobility was connected to a robotic hand prosthesis by means of MI [26]. After five months of training, the subject acquired some control over the prosthesis. Interestingly enough, shorter training periods have also been reported to be successful [27]. Moreover, several BCI paradigms have been combined simultaneously, for example to control a robot arm that simulates an upper human joint with two degrees [28]. This application used MI to control the gripping function and steady-state visual evoked potentials (SSVEP) for the joint control.

In [29,30], extensive reviews of state of the art exoskeletons developed for the rehabilitation of the upper limb are presented. Some exoskeletons for upper limb rehabilitation are already commercially available, i.e., Aupa, JACE S600, JACE S603 and Armeo^®^ Spring. The main disadvantages of these devices are that the anthropometric measures, on which their design are based, may not correspond to the population in which they are to be applied, and also that their high cost of acquisition and maintenance make them inaccessible to most institutions. These limitations have motivated the research carried out in this study in order to produce a customized, low-cost rehabilitation device.

Motor imagery based BCI (MI-BCI) has been used to carry out robotics-assisted rehabilitation in several studies [31,32]. In one of them [32], a group of patients who underwent standard robotic rehabilitation was compared with another group using MI-BCI, the latter of which achieved superior performance. A combination of MI-BCI and conventional physical therapy [26] to rehabilitate patients who had suffered a stroke has also shown that patients treated with MI-BCI improve more than those treated with random BCI.

MI-BCI has proven its potential to help in the rehabilitation process, but its learning curve is steep and weeks of training are required to gain a satisfactory level of control [33]. Many people only achieve a deficient level of BCI control, even when extended training is provided, a phenomenon that has been labelled ‘BCI illiteracy’ [34].

Several MI strategies have been studied, such as hand and foot movements, mental mathematical operations, and mental rotation of objects [35,36,37,38]. MI strategies based on mental imagination of limb movements seem to be more appropriate for limb rehabilitation [39]. Pfurtscheller et al. [26] used brain oscillations to control an electrically driven hand orthosis for restoring hand grasp function. The subjects imagined left versus right hand movements, left and right hands versus no specific imagination, and both feet versus right hand, and achieved an average classification accuracy of approximately 65%, 75% and 95%, respectively. Which particular motor imaginations allow for a better control, though, remains an open issue. In this paper, different motor imagination strategies are compared.

This work implements a low-cost robotic rehabilitation assistance system. The subjects’ will to move their arms is interpreted by analyzing their motor imagery by means of processing the EEG signal from the motor cortex. The rehearsed movements of the users are decoded from their MI and then translated into the real movement of the rehabilitation device. BCI performance in the control of the rehabilitation device with different motor imagery tasks is assessed. For this purpose, two experiments were planned. In the first experiment, action instructions (imagined movement of hands or feet) are compared with non-action instructions (imagined motionless hands or feet); while in the second experiment, two different action strategies (right/left hand movements versus hand/feet movements) are compared.

In the second section of this paper, the design and construction of the rehabilitation guide is presented. The guide control system based on a motor imagery BCI is explained. The experiment procedure is described as well as the different hypotheses tested. In Section 3 and Section 4, results of the experiments are shown and discussed. Finally, some conclusions are drawn.

## 2. Materials and Methods

### 2.1. Rehabilitation Guide

**Figure 1:**
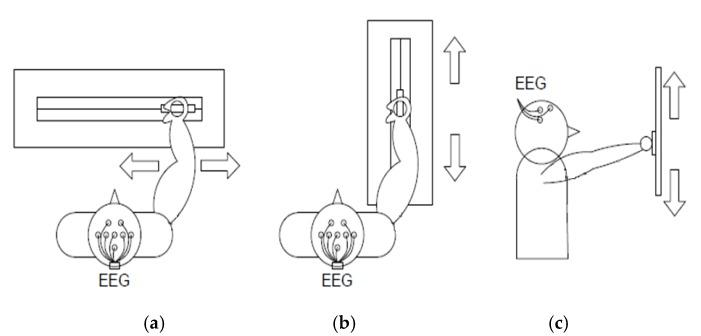
Rehabilitation guide operation modes. EEG = electroencephalogram. (**a**) Transversal movement; (**b**) longitudinal movement; (**c**) vertical movement.

The first objective of this work was to design a low-cost robotic device useful for the rehabilitation of arm movements in affected patients. The system was designed to enable various types of rehabilitation depending on the placement of the guide. Thus, it should be possible to rehabilitate vertical, horizontal, and longitudinal movements as shown in Figure 1. The position, speed and direction of a carriage attached to the guide are controlled. The patient places his or her hand on the mobile carriage to rehabilitate the arm’s motor function.

The structure of the guide is made up of a metallic aluminum assembly with a square section of 4.5 cm and a longitude of 110 cm. On this, a carriage made by three-dimensional (3D) printing is mounted, which can slide longitudinally along the assembly. On this car, wrist attachments are fixed that adapt to the subject’s arm in order to perform the rehabilitation.

The transmission system of the mobile carriage consists of a drive gear (double helical driver with 21 teeth) that rotates with the motor shaft. A double helical drive gear with 10 teeth rotates around the driving pulley and drags the carriage on a belt. The drive pulley has a radius of 25.21 mm and 0.254 mm teeth and the pulley on the right end of the guide is the same as the drive.

The relationship of movement between the carriage and the motor revolutions is obtained from Equation (1).

(1) $$\frac{21}{10}\cdot 2\pi \cdot 25.464=336.46\mathrm{mm}/\mathrm{rev}\;$$

The Direct Current (DC) motor that drives the gear system has a nominal supply voltage of 12 V and in nominal operation can vertically raise 3 kg (as measured on the guide carriage). This motor has a coupled reducer and an incremental encoder. The encoder’s output determines the carriage’s direction of movement and number of movement steps. A limit switch was placed on the extreme left end of the guide to enable the system to home at the beginning of the program execution and so to have a carriage position reference.

To enable the carriage to move in both directions, the motor is controlled from a driver or H-bridge controlled by digital signals from the PC. Specifically, the integrated BD6231F-E2 is a H-bridge that also enables control of the output voltage through pulse width modulation (PWM) so that with the same component, it is possible to control movement speed and direction. A LabVIEW program controls the driver moving the carriage via the NI-6210 acquisition card. The carriage movement is limited at the beginning of every trial according to the desired span for every user.

**Figure 2:**
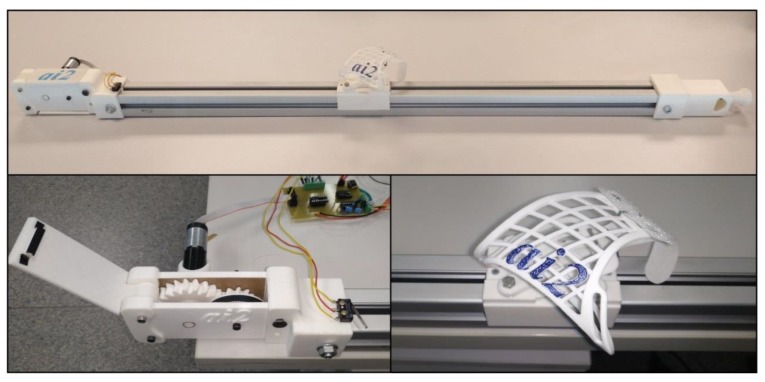
Rehabilitation guide.

The rehabilitation guide is powered from batteries to ensure a stable voltage, to facilitate the portability, and to reduce the risk of patient electrocution. One of the design requirements of this work was the construction of a low-cost rehabilitation device, and for that reason, many of the components of the guide have been 3D-printed (Figure 2).

To begin with the rehabilitation, the user places his or her hand on a wristband, which has been specifically molded for the patient, and is made of a thermoplastic insole. The user then must try to move the carriage towards the right or left, backwards or forwards, or up or down, depending on the rehabilitation exercise and, hence, on the position of the guide. These different configuration options allow for the same guide to perform different rehabilitation exercises so that it can be adapted to the requirements of each patient.

Once the user begins to try (although their mobility in this joint could be very limited or even fully absent), by processing the EEG signals, it is possible to detect the direction of the user’s intended movement and this information is used to start the guide motor and to move its articulation accordingly. The user therefore obtains a movement feedback that reflects his or her intention. The guide’s motor and transmission system prevent any actual movement of the carriage if no user intention is detected.

### 2.2. EEG-Based Control

EEG signals from the motor areas of the patient are registered to control the guide movements. These signals are processed and a control action (which can be movement in one direction, in the other, or stop) is sent to the guide. The speed of movement is regulated through PWM. This speed control is external to the patient and performed by the person responsible for the rehabilitation.

**Figure 3:**
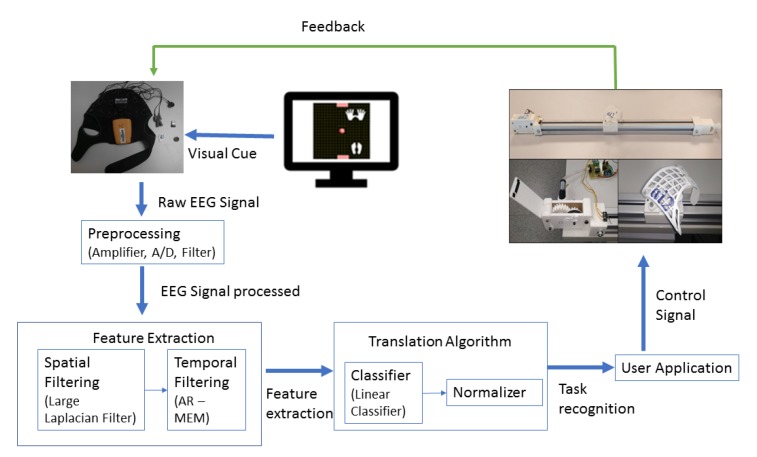
Motor imagery brain–computer interface (MI-BCI) for the rehabilitation process.

Motor imagery is used to control the rehabilitation guide movements. The subject being monitored is asked to think of a type of movement to detect variations in the EEG related to this task with the electrodes placed in the sensorimotor areas of the brain. The concept of building a BCI with motor imagery lies in creating a computer algorithm that detects the changes in brain waves associated with the patient’s movement intention and translates these changes into computer signals [40,41]. The guide then moves according to the user’s intention, providing visual and somatosensory feedback (Figure 3).

**Figure 4:**
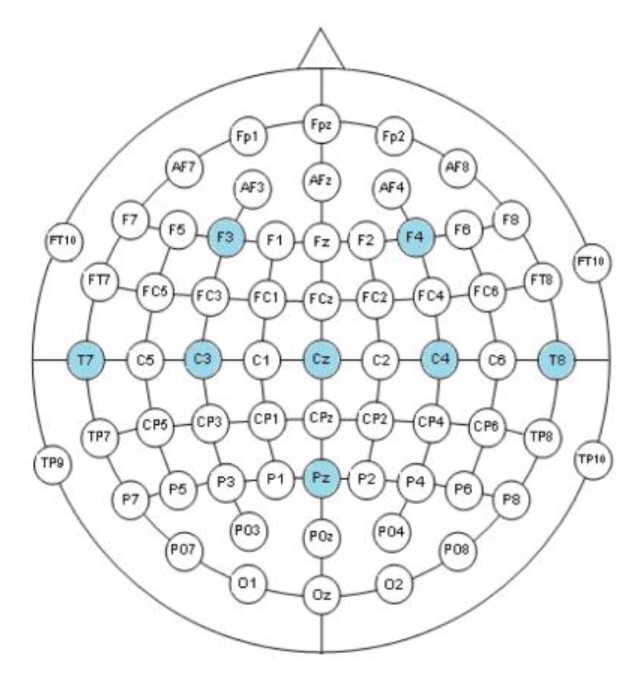
Registered EEG channels.

Several factors have been taken into account when selecting hardware and software components for the EEG-based BCI. Given the interest in a portable and compact solution for BCI control, the Enobio digital amplifier [42] from Neuroelectrics was selected to acquire the EEG signals. The Enobio amplifier was developed for BCI research. It was chosen because of its wireless technology and dry electrodes that facilitate the experimental setup. The EEG signal was acquired through channels F3, F4, C3, Cz, C4, T7, T8 and Pz around the sensorimotor areas according to the standard 10–20 electrode location system (Figure 4). Ground and earth electrodes were placed in the subject’s earlobe. The EEG signal was recorded using a sampling rate of 500 Hz and band-pass filtered between 2 and 100 Hz with an activated notch filter at 50 Hz. The sampled and amplified EEG signal is then sent to the computer via Bluetooth.

To implement rehabilitation guide control, the open-source BCI software BCI2000 (Wadsworth Center, New York State Department of Health in Albany, New York, USA) was used [43]. The carriage movement was controlled after the cursor movement on the computer screen in the cursor task of BCI2000 [44]. The cursor task is based on the modulation of mu and beta rhythms and allows the participants to control the position of a cursor on the computer screen. The participants’ intentions affect the cursor position by means of imagining motor actions. In this study, the participants had to follow the instructions received to direct the cursor towards a bar that could appear in different parts of the screen. Being able to direct the cursor and to reach the bar in a pre-specified time interval was considered as a successful attempt. The participants had to control the direction in which the cursor was moving in order to reach the bar. EEG signal processing, feature extraction and classification followed the procedure described below [22,45,46,47].

The EEG channels are spatially filtered to improve the signal-to-noise ratio. A large Laplacian filter is chosen as shown in Equation (2) [48]:

(2) $$C_{out}=\;\sum_{k=1}^{n}C_{k}W_{k}$$

where *C_out_* is the electrode to be analyzed, *C_k_* is the electrode k signal, n is the total number of electrodes and *W_k_* is the weight of electrode k. In this study, the filtered channels C3, CZ and C4 are chosen as output channels according to the weights shown in Table 1. These channels are located on the motor somatosensory cortex areas corresponding to the right hand, feet and left hand respectively.

BCI2000 uses an AR model to estimate the sensorimotor rhythm amplitudes for control according to Equation (3) [49]:

(3) $$Y_{t}=\sum_{i=1}^{i=p}a_{t-1}Y_{t-1}+e_{t}$$

where *Y_t_* is the predicted signal at time *t*, *a* is a vector of *p* coefficients, and *e* is the prediction error. *a* is a vector of estimated filter coefficients for an all-pole model of order *p*, for which the power spectrum is found as shown in Equation (4).

(4) $$\hat{P}\left(e^{jw}\right)=\frac{1}{\left|1-\;\sum_{k=1}^{r}a_{p}\left(k\right)e^{-jkw}\right|2\;}$$

In order to evaluate the model coefficients, BCI2000 employs the Maximum Entropy (or Burg) method [50]. BCI2000 is configured to calculate the power in adjacent bins of 3 Hz width. Within each bin, the power is estimated at evenly-spaced intervals and averaged. In this case a 3 Hz bin from 9–12 Hz with 11 evaluations finds the power in 0.2 Hz intervals (Equation (5)).

(5) $${\hat{P}}_{9-12}=\;\sum_{wI=9,\;0.2}^{12}\left[\left|1-\sum_{k=1}^{r}a_{p}\left(k\right)e^{-jkwi}\right|{}^{-}2\right]$$

Each of the three output channels then have a number of binned power spectrum amplitudes. The linear classifier subsequently translates these features into output control signals. Its output is normalized with respect to mean and variance and used to determine a virtual cursor movement in the computer screen [43].

Since the cursor task is implemented in BCI2000 and the rehabilitation guide control program was written in LabVIEW [51], a gateway between these software tools was developed. The connection between LabVIEW and BCI2000 is based on the UDP protocol where BCI2000 acts as the server and LabVIEW acts as the client.

### 2.3. Participants

Although the ultimate goal is the therapeutic use of the proposed rehabilitation device, healthy non-disabled participants have been selected for this study in order to validate the security and feasibility of the rehabilitation guide and to determine the most convenient MI strategies.

All procedures performed involving human participants were in accordance with the ethical standards of the Universitat de València research committee and comparable ethical standards. Informed consent was obtained from all individual participants included in the study.

The participants were students from the Universitat de València. They each fulfilled several questionnaires and tests and performed two different BCI tasks. None of them had previous experience with BCIs. A medical history of epilepsy or the intake of psychoactive drugs were exclusion criteria for this experiment and none of the participants were rejected for these causes.

Two different experiments concerning BCI task MI strategies have been carried out. Fifty participants (10 males, 40 females) with a mean age of 20.18 years (standard deviation (SD) = 3.04) took part in the first experiment. For the second experiment, 127 participants (11 males, 116 females) with a mean age of 18.01 years (SD = 0.59) were selected. Data from participants who did not complete the whole protocol have been excluded. The initial number of participants in the first experiment was 90 and in the second experiment 183.

In order to investigate correlations between BCI performance and user traits, the participants went through a demographic survey and several psychological tests. An initial questionnaire designed by us explored some participants’ traits, psychological variables and habitually performed daily activities (physical exercise, video games, music training etc.). These activities have been hypothetically related to the ability to manage a BCI device [35]. Results regarding these psychological traits and their correlation with BCI performance were shown in [52].

### 2.4. Experimental Procedure

The session in each experiment lasted for approximately 60 min and was organized as shown in Table 2.

Preparation: Participants were informed about the experiment procedure and signed the informed consent. The Enobio helmet was properly placed on their heads [53]. The task instructions were provided during the habituation period.

Relaxation: Immediately before starting the tests, participants performed a Jacobson’s progressive facial relaxation procedure guided by recorded verbal instructions for 180 s. The role of this relaxation procedure was to induce a relaxed state in the participants. It was conducted because tension has been shown to correlate negatively with motor imagery BCI performance [40].

BCI tasks: Each participant performed two control tasks that differed in the MI strategy. The task order was randomized between subjects.

**Figure 5:**
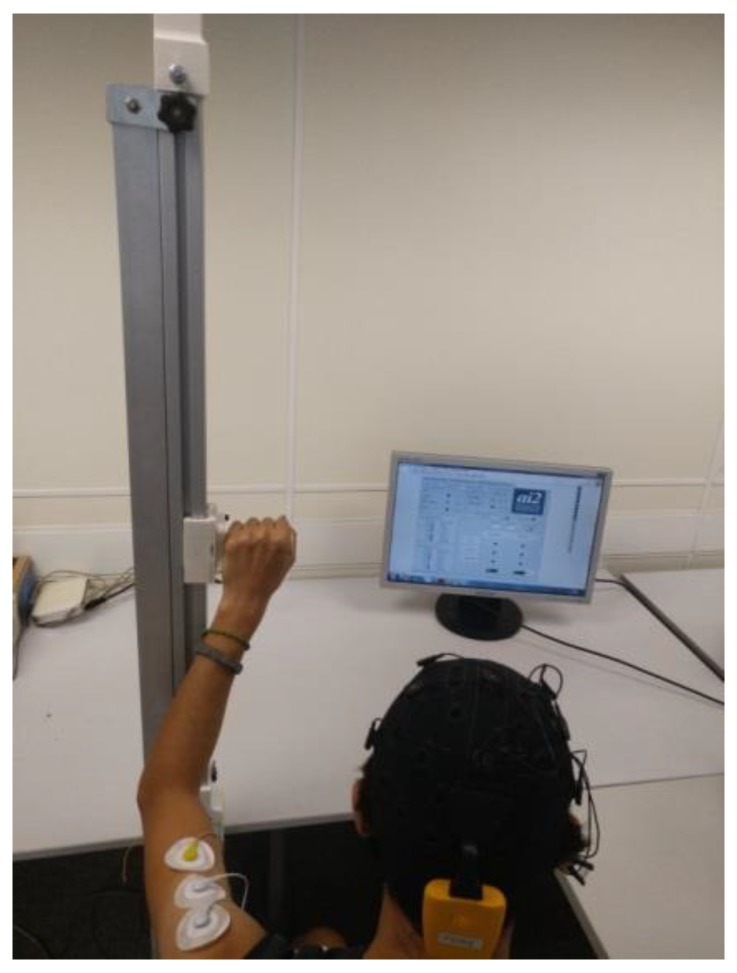
Vertical movement rehabilitation.

In the first experiment, a vertical arm rehabilitation was rehearsed (Figure 5). The guide moved the subject’s arm up and down according to the MI performed. In the first strategy, the users followed an action–relaxation instruction and in the second strategy, they followed an action–action instruction. For the action–relaxation instruction (hand/relax task (HRT)), subjects had to purport moving their hand to move the guide up and to relax if they wanted to move it downwards. In the action–action task (hand/feet task (HFT)), they had to purport moving their hand to move the guide up. If they wanted to move the guide down, they were instructed to imagine that they were stretching their feet.

To provide a task reference and feedback to the subjects, a cursor task was shown on the computer screen [43]. Targets appear on the screen and participants were asked to imagine the instructed movements to move the cursor towards the targets.

Six 150 s tests were performed by each participant (three per type of task), divided into 20 s trials. In each trial, the cursor was visible for a maximum of 20 s, during which they could succeed (the cursor reached the target) or fail (the cursor did not reach the target). In both cases, a new trial was subsequently initiated. If the 20 s period finished without the cursor reaching either side, a new trial was started. Henceforth, the number of trials for each participant depended on the time they spent in each trial. The carriage in the rehabilitation guide moved after the cursor position between two prefixed limit positions. They were asked to imagine repetitive movements as long as they wanted the cursor, and the guide, to keep on moving to reach the target.

In the second experiment, a horizontal arm rehabilitation was rehearsed. The guide moved the subject’s arm to the right and to the left according to the MI performed. Two different action–action tasks were compared. The previous HFT was compared with a right hand/left hand task (RLT) for moving the guide in a horizontal position. In this RLT, targets appear on the right and left sides of the screen and participants were asked to imagine right-hand movements to direct the cursor to the right, and the opposite for the left side (Table 3).

After performing the BCI tasks, the participants completed a test to evaluate their subjective experience of the experimental procedure.

To analyze the results, IBM SPSS Statistics software v. 16.0 was used. *t*-tests were performed for related samples and paired samples, and univariate variance analyses have been performed.

## 3. Results

**Figure 6:**
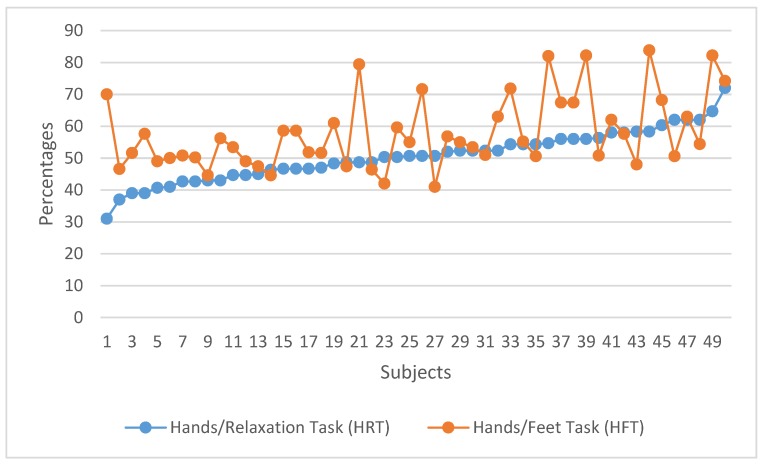
Individual task performance in HRT vs. HFT.

**Figure 7:**
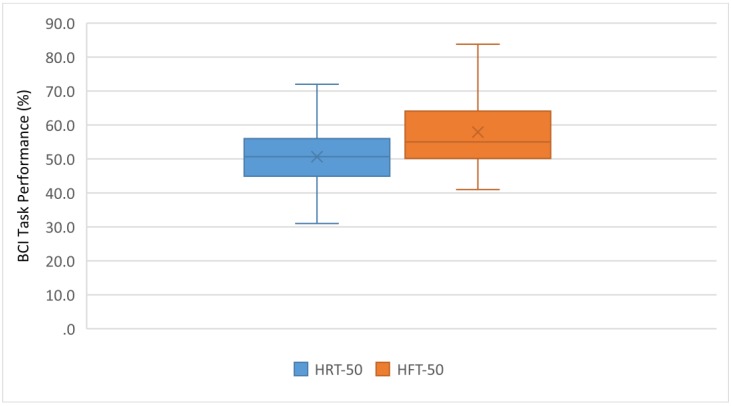
Task performance average in HRT vs. HFT.

In the first experiment, an action–action strategy (HFT) was compared with an action–relaxation strategy (HRT) in the MI-BCI task. Figure 6 shows the individual task success comparing the HRT and HFT control strategies. In Figure 6, results for all subjects are ordered according to their performance at the HRT. This ordered structure facilitates the comparison between ‘good’ and ‘bad’ performers for each condition and it shows the variability of the data. Figure 7 shows the group performance averages in each strategy. These percentages are averaged over the three trials in each condition.

Figure 6 and Figure 7 show that the HFT strategy produced a better performance: 76% of the participants (38 out of 50) achieved better results than with HRT. A statistically significant difference between both strategies was found: the control on HRT trials was significantly lower than on HFT: *t* (49) = −4.667, *p* < 0.001.

As expected, average task performance was low for subjects without previous training in MI-BCI. For the HRT, no learning was observed among the participants (operationalized as an increase in the percentage of successful attempts between the first and the last trial); the difference between both attempts was not significant. For the HFT, though, an improvement was observed between the first and the third trial (*t* = −2.425; *p* = 0.010).

As shown in the results of this first experiment, for most subjects, it was easier to use an action–action motor imagery strategy than an action–relaxation one.

**Figure 8:**
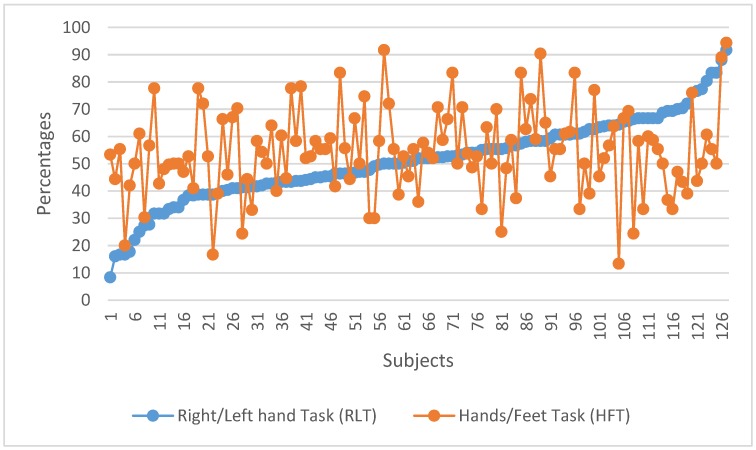
Individual task performance for RLT vs. HFT.

**Figure 9:**
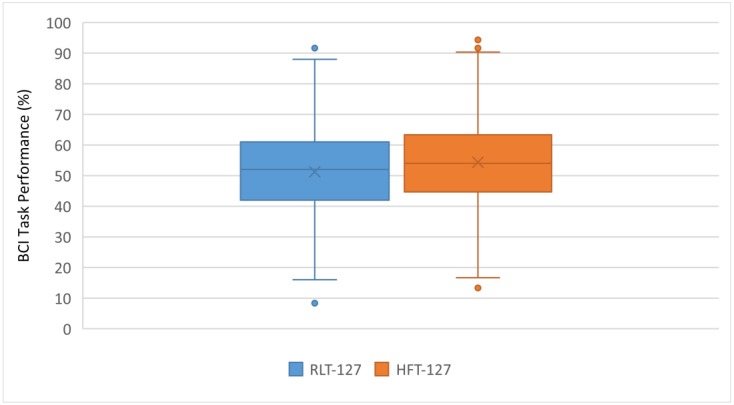
Task performance average for RLT vs. HFT.

To compare different action–action motor imagery strategies, a second experiment was designed with a different group of participants (*n* = 127). In this experiment, the HFT and RLT strategies to control the horizontal movement of the rehabilitation guide were compared. Figure 8 shows the individual task success comparing both strategies. Individual results for all subjects are ordered according to their performance in the RLT. Figure 9 shows the group performance averages for each strategy.

Figure 8 and Figure 9 suggest that the general performances with both action–action strategies (RLT and HFT) are similar. This was confirmed with a mean difference hypothesis test that showed no statistically significant differences between both strategies.

A unifactorial inter-group ANOVA was performed using the group variable as the independent variable (first group, *n* = 50 and second group, *n* = 127) and the HFT performance index as dependent variable. Levene’s test indicated compliance with homocedasticity (F_(1,175)_ = 3.597; *p* = 0.060). Regarding the results of the ANOVA, they showed the absence of statistically significant differences between both groups in the HFT performance variable (F_(1,175)_ = 2.074; *p* = 0.152; $\eta_{P}^{2}$ = 0.012; 1-β = 0.299). Consequently, the averages in both groups did not show the existence of greater efficacy in one group (*n* = 50; mean = 57.91; SD = 11.30) with respect to the other (*n* = 127; mean = 54.39; SD = 15.80). 

Table 4 shows the results of the initial questionnaire completed by the participants.

Feedback was obtained from the participants about their experience in wearing the EEG cap in the BCI tasks in relation to experienced pain and comfort (Table 5 and Table 6).

## 4. Discussion

This study presents a low-cost rehabilitation device controlled via an MI-BCI system. The construction of the guide with rapid prototyping techniques such as 3D printing makes it compact, lightweight and economical. The device for holding the patient’s hand to the guide was printed on a rigid material and a thermoforming process was applied that allows a customized fit. The use of open-source BCI software, such as BCI2000, also contributes to an easy replicability of the prototype.

The proposed robotic guide permits different rehabilitation exercises (shoulder and elbow in vertical, horizontal and longitudinal movements), depending on its positioning in relation to the subject. The range and speed of the rehabilitation movements of the guide are adaptable to each patient.

This research has been subject to several limitations. Although a higher number of sessions would have been convenient, our participants were students enrolled in a course taught by one of the authors and their participation was a part of the course’s program. The end date of the course, as well as the fact that the BCI-related practice was a part of the syllabus, precluded us from the possibility of performing further experimental sessions. An additional limitation relates to the present prototype, which only allows for unidimensional movements. We are currently working on a robotic device with similar characteristics that will hopefully allow for the rehabilitation of two-dimensional and three-dimensional movements.

Mean contrasts were performed for independent groups using Student’s t-test for all the dichotomous variables of the initial questionnaire versus the performance of each subject in the BCI tasks. No statistically significant differences were detected in any of the variables except for Q4 (*t* (127) = 2.8981; *p* = 0.005; SD = 7.62).

From the data of the opinion questionnaire about the experiment, it can be concluded that the experience of the subjects has been good, considering that almost 95% of the respondents reported that wearing the EEG helmet with dry electrodes produced little or no discomfort (Table 5). Similarly, approximately two-thirds of the participants estimated that they could wear the EEG helmet for up to two hours continuously (Table 6). It can be concluded that the use of dry electrodes and wireless EEG signal transmission made the experimental setup tolerable and even comfortable.

In this work, several MI strategies to control the device in different rehabilitation exercises have been compared. The fact that an action–action MI strategy provides better results than an action–relaxation MI strategy can be related to the nature of the EEG signals and their distribution over the scalp. Switching between two different MI tasks that are associated to opposite sides of the body (i.e., left hand vs. right hand) triggers the activation of different areas of the motor cortex. This fact makes it easier for the classification algorithms to detect the changes in the EEG data in an action vs. action condition than when it is an action vs. rest task, in which there are just variations of EEG power in a single area of the brain.

In this study, an active BCI paradigm was used where the user performed mental tasks voluntarily (thinking about the movement of the right hand, left hand, feet, or relax). Motor imagery was well accepted by the users because it provided a sense of agency compared to other reactive or passive paradigms [54]. Reactive BCI paradigms such as SSVEP occur when the user’s brain reacts to external stimuli (visual, auditory or tactile). In passive BCI paradigms, the user’s mental state is analyzed in real time such as in workload estimations.

The MI rehabilitation paradigm applied in this study is not limited to a specific type of patient condition. According to similar studies [8,55,56], it could be used for post-stroke treatment, spinal cord injury (SCI) patients, trauma, etc. Moreover, as [57] have shown, by simultaneously combining motor imagery and action observation when compared to simply observing the action, we see an enhanced corticomotor excitability that might result from the activation of mirror neurons. This promising conclusion highlights that the results obtained in this study might be applied to the rehabilitation of medullary-injured patients.

## 5. Conclusions

An MI-BCI controlled low-cost robotic system for the rehabilitation of arms has been designed. The feasibility and safety of the system has been tested with an extended healthy population. All subjects could perform the intended arm movements. The rehabilitation guide has been controlled by the users according to his or her intentions with MI-EEG. Validation of the BCI control of the rehabilitation guide by a healthy population, as well as the selection of the most effective MI strategy, were necessary steps before applying it to patients.

These results show a better performance with an action–action MI strategy than with an action–relaxation MI strategy. A statistically significant difference between the two action–action motor imagery strategies was not found.

Once the feasibility and safety of the robotic guide for arm rehabilitation has been checked with healthy subjects, the next stage would be to apply the system to patient populations. Since MI-BCI has been used in previous studies with post-ictus patients and the rehabilitation guide does not require active muscle exercise, the authors are confident that this system may become a useful tool in the rehabilitation of arm movements.

## Figures and Tables

**Table 1:** Laplacian filter coefficients. *C_k_* = electrode k signal.

				*C_k_*				
*C_out_*	F3	F4	T7	C3	CZ	C4	T8	PZ
C3	−0.25	0	−0.25	1	−0.25	0	0	−0.25
CZ	−0.2	−0.2	0	−0.2	1	−0.2	0	−0.2
C4	0	−0.25	0	0	−0.25	1	−0.25	−0.25

**Table 2:** Experimental procedure.

Step	Time (Minutes)	Activities
Preparation and information	15	Filling out consent form General information (posture, stop the experiment) Initial questionnaire Electrode placement
Relaxation	5	Jacobson’s progressive facial relaxation procedure
MI-BCI tasks	30	BCI tasks
Opinion questionnaire	5	Experiment evaluation test
Experimental end	5	

**Table 3:** BCI tasks.

Experiment	Paradigm	Task	Visual Cue/Description
1 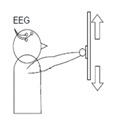	Hand movement versus relax (HRT)	“↑” imagine opening and closing hand “↓” no movement and relax	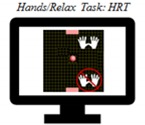
1 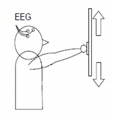	Hand versus feet movement (HFT)	“↑” imagine opening and closing hand “↓” imagine both feet flexion	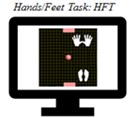
2 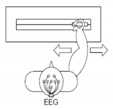	Right hand versus left hand movement (RLT)	“←” imagine opening and closing left hand “→” imagine opening and closing right hand	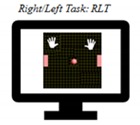
2 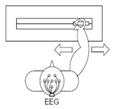	Hand versus feet movement (HFT)	“←” imagine both feet flexion “→” imagine opening and closing hand	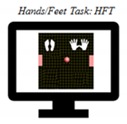

**Table 4:** Initial questionnaire.

Questions	Answers	Percentage
Q1: Dexterity	Right	92.6
	Left	7.4
Q2: Do you play any musical instrument?	Yes	17.1
	No	82.9
Q3: Do you consider yourself a bilingual person?	Yes	70.9
	No	29.1
Q4: Did you sleep well last night?	Yes	64.2
	No	35.8

**Table 5:** Pain perception.

Discomfort	None	Little	Moderate	A Lot	Too Much
Participants (%)	88 (49.7%)	79 (44.6%)	10 (5.7%)	0 (0%)	0 (0%)

**Table 6:** Comfort perception.

Tolerance Time	<1 h	1–2 h	2–4 h	Almost All Day	All Day
Participants (%)	30 (17.0%)	79 (44.6%)	29 (16.4%)	29 (16.4%)	10 (5.6%)

## Abbreviations

The following abbreviations are used in this paper:

AMT	Active motor training
BCI	Brain–computer interface
EEG	Electroencephalography
HFT	Hand/feet task
HRT	Hand/relax task
MI	Motor imagery
PWM	Pulse width modulation
RLT	Right hand/left hand task
SCI	Spinal cord injury
SPSS	Statistical Package for the Social Sciences
SSVEP	Steady-state visual evoked potentials
UDP	User datagram protocol
